# Virtual assessment, training, and evaluation of clinical laboratory technologists amidst peak Covid-19 pandemic: An observational study

**DOI:** 10.5937/jomb0-33962

**Published:** 2022-10-15

**Authors:** Sibtain Ahmed, Abid Muhammad Abbas, Farhat Jahan, Tomris Ozben

**Affiliations:** 1 Aga Khan University, Department of Pathology and Laboratory Medicine, Section of Clinical Chemistry, Karachi, Pakistan; 2 Akdeniz University, Department of Medical Biochemistry, Antalya, Turkey

**Keywords:** quality control, technologist, Covid-19, Unity Real Time, laboratory, clinical chemistry, kontrola kvaliteta, tehničar, Covid-19, Unity Real Time, laboratorija, klinička hemija

## Abstract

Medical technologists are considered a neglected group when it comes to academic interventions. We developed and implemented an educational intervention and assessment for the technologists based on an online questionnaire as a pre-test consisting of questions related to knowledge (n=5), attitude (n=3), and practices (n=4) of daily internal quality control (QC) monitoring via Google Docs survey tool. This study served multiple purposes. It allowed keeping the technologists engaged during the peak of the COVID-19 pandemic while also improving the knowledge, attitude, and practices about the internal quality control using Bio-Rad Unity Real Time (URT) QC software. Subjects were graded based on the scores they received out of 100 (0-60 = poor; 61-79 = good; 80-100 = excellent). Training materials, i.e., a set of 5 videos every week via e-mail, were circulated. A voice-over PowerPoint presentation was also shared for easy comprehension. This activity was repeated after one month. A post-test was administered to assess the improvement. The study results show significant improvement in the technologists' performance after the intervention.

## Abstract (sr)

Medicinski tehničari se smatraju zapostavljenom grupom kada su u pitanju akademske intervencije. Razvili smo i implementirali edukativnu intervenciju i procenu za tehničare na osnovu onlajn upitnika kao pre-testa koji se sastoji od pitanja vezanih za znanje (n = 5), stav (n = 3) i prakse (n = 4) svakodnevnog praćenja interne kontrole kvaliteta (QC) putem alata za anketiranje - Google dokumenata. Svrha ove studije je bila višestruka. Omogućila je angažovanje tehničara u jeku pandemije COVID-19, a istovremeno je poboljšala znanje, stav i praksu o internoj kontroli kvaliteta koristeći Bio-Rad Uniti Real Time (URT) softver za kontrolu kvaliteta. Subjekti su ocenjeni na osnovu ocena koje su dobili na skali do 100 (0-60 = loše; 61-79 = dobro; 80-100 = odlično). Materijali za obuku su distribuirani putem elektronske pošte - set od 5 video zapisa svake nedelje. Podeljena je takođe i PowerPoint prezentacija radi lakšeg razumevanja. Ova aktivnost je ponovljena nakon mesec dana. Sproveden je i post-test da bi se procenilo poboljšanje. Rezultati studije pokazuju značajno poboljšanje učinka tehničara nakon sprovedene intervencije.

## 55

The utility of medical technologists in the laboratory has been well-established since the 1930s [1]. Specifically, in clinical pathology, medical technologists play a much more important role than anatomical and other aspects of pathology [1]. A quality medical laboratory is an important part of the healthcare system, and quality control (QC) is one of the most pivotal pillars of laboratory testing [2] [3]. Historically, technologists are often neglected in terms of training and academic progression [4].

Dissemination of new knowledge to medical technologists is an integral part of laboratory practice [5]. Educational interventions in the field of laboratory management have proven to be effective. However, the effect of intervention disappears over time unless additional interventions are provided to sustain performance [6]. The management of internal QC of the College of American Pathologists (CAP) accredited Clinical Chemistry laboratory at the Aga Khan University Hospital (AKUH) has been evaluated to promote an efficient and comprehensive process of internal QC management. Bio-Rad Unity Real Time (URT) QC software is used to monitor internal QC. The URT software is configured and consolidated in the hospital's Integrated Laboratory Management System (ILMS). URT software provides statistical review, documentation, and analysis across all facets of the laboratory. It is networkable to include all areas of the laboratory. The software analyses the QC data for laboratory staff to complete their QC tasks as required and also allows for external peer-group analysis [7]. However, substantial differences and deficiencies were encountered repeatedly on review by the sectional QC coordinator.

The Coronavirus Disease 2019 (COVID-19) impacted healthcare delivery at all levels [8]. Specific focus and resources have been applied to diminish the effects on physicians, patients, and healthcare systems with little attention to delineating and addressing the issue of medical technologists [9].

This study aims to assess the knowledge and practice of medical technologists by online QC monitoring using Bio-Rad Unity Real Time Software (URT) and to keep the technologists engaged during the peak of the COVID-19 pandemic.

The study was performed at the Section of Clinical Chemistry, Aga Khan University Hospital (AKUH), Karachi, Pakistan, from June to September 2020. The study participants comprised three tiers of clinical scientists working in the laboratory, ranging from the senior-most cadre of charge technologists, senior technologists, and technologists. A total of 20 lab technologists were randomly selected with equal gender distribution, and the functional grades covering different tiers of laboratory staffing are enlisted in Table 1.

**Table 1:** Demographic distribution of study participants

Variables		Number <br>(n)	Percentage <br>(%)
Gender	Female	10	50
	Male	10	50
Designation	Senior Technologist	9	45
	Technologist	9	45
	Charge Technologist	2	10

An online questionnaire-based pre-test consisting of questions related to knowledge (n=5), attitude (n=3), and practices (n=4) of daily internal QC monitoring via URT was administered via Google Docs survey tool as shown in Table 2. Electronic consent for participation was acquired at the start of the survey. The study was approved by the sectional and departmental Quality Management Committee, Department of Pathology and Laboratory Medicine, AKUH (CA-01/2020). One laboratory medicine consultant and a QC in charge at the section designed the questionnaire which was validated as a pilot with 2 postgraduate trainees who were part of this initiative team. Keeping the hectic bench side work schedule that had vastly exalted during the peak of the pandemic and inherent time constraints in mind, the questionnaire was structured in such a way that it did not consume more than 15 minutes of a participant's time. Subjects were graded based on the scores they received out of 100 (0-60 = poor; 61-79 = good; 80-100 = excellent).

**Table 2:** Study Questionnaire employed pre- and post-intervention

Knowledge	Are you able to show LJ charts on URT screen and print the charts?	
	Are you able to show all point data, summary data, statistics, and LJ on one screen?	o Yes<br>o No
	Are you aware of the »Lab navigation tree, panel navigation tree, and Instrument navigation tree« options in URT?	o Yes<br>o No
	Floating mean can be defined as?	o Monthly mean<br> o Cumulative mean<br> o Establish mean<br> o None of the above
	Which QC parameter is helpful to review monthly QC in URT?	o Appropriate use of QC rules for a measuring method<br> o Median value of patient test result <br>o Z score <br>o Six monthly cumulative CV value
Attitude	According to your experience, is URT better than a traditional paper-based QC?	o Yes<br> o No
	In-charge asked you to check the control trend of calcium between Advia 1 and Advia 2. What should you do?	o You would take control LJ prints of two instruments from URT.<br> o You would show control LJ of two instruments on URT.<br> o You would check control LJ on URT and give feedback to a pathologist.
	Bench review and supervisor review of controls run on any instrument should be done in the following order in URT	o Testing technologist → Senior Technologist →Bench in-charge → Resident → Pathologist/designee → Section head <br>o Testing technologist → Bench incharge → Pathologist/designee <br>o Testing technologist à Bench in-charge → Resident → Pathologist/ designee <br>o Bench in-charge → Pathologist/ designee
Practice	Are you able to use your control-related navigator?	o Yes, I can use all of them <br>o I can use only one of them <br>o I can use two of them <br>o I don’t know how to use
	Are you able to add comments, corrective actions in URT?	o Yes <br>o No <br>o I can add corrective actions but don’t know how to add comments
	One the 22nd day, when technologists observed one control outside 3SD, what should be done after taking corrective actions?	o Check URT and, if the control result found acceptable, then issue the patient report <br>o All corrective actions documented in URT under the option of »comments. <br>o All corrective actions document in URT under the option of »action.«
	Due to technical issues, results of (multiqual) control used for 12 analytes were not transmitted in URT. What should a technologist do?	o Inform his/her senior and document in the file. <br>o Enter all results in URT one by one. <br>o Enter all results in a single option in URT

Training materials, i.e., a set of 5 videos every week via e-mail, were circulated. A voice-over PowerPoint presentation was also shared for easy comprehension. This activity was repeated after one month. A post-test was administered to assess the improvement.

Statistical analysis was performed using Microsoft Excel 2013 and Statistical Package of Social Sciences (SPSS) version 19. Frequency and percentages were calculated for gender and designation. Paired Samples t-test was performed to evaluate whether there is statistical evidence that the mean difference between paired observations between pretest and post-test with an intervention administered between the two time points is significantly different. Two-tailed p-values < 0.05 were considered significant.

The mean pre-test score ± standard deviation (SD) was 42.5±14.82. The grades received in pretest were Excellent (n=0), Good (n=1), Poor (n=19). The mean post-test score ± SD was 81.5±15.31. The grades received in the post-test were Excellent (n=15), Good (n=1), Poor (n=4). A statistically significant difference was noted between the pre and post-test results (p<0.001). The results and the differences in pre-and post-test performance are detailed in Table 3.

**Table 3:** Comparison between Pre and Post audit Response.

Technologist	Pre-audit	Grade	Post<br>Audit	Grade	p-value
1	50	Poor	90	Excellent	0.000
2	40	Poor	100	Excellent	
3	40	Poor	50	Poor	
4	40	Poor	100	Excellent	
5	60	Poor	100	Excellent	
6	50	Poor	80	Excellent	
7	60	Poor	80	Excellent	
8	60	Poor	100	Excellent	
9	50	Poor	90	Excellent	
10	30	Poor	80	Excellent	
11	20	Poor	60	Poor	
12	50	Poor	80	Excellent	
13	50	Poor	80	Excellent	
14	70	Good	70	Good	
15	30	Poor	100	Excellent	
16	30	Poor	80	Excellent	
17	20	Poor	60	Poor	
18	20	Poor	60	Poor	
19	30	Poor	90	Excellent	
20	50	Poor	80	Excellent	

The results of our study demonstrated significant improvement in medical technologists' performance post-intervention. Medical technologists are essential members of the overall healthcare provision team. The knowledge of modern laboratory techniques is volatile and rapidly evolving [5]. Periodic educational interventions are necessary to ensure better QC and also equip technologists with the latest information in their specific field. When it comes to academic growth and development, medical technologists are a neglected group amongst healthcare professionals [4]. It is prudent to design educational interventions specific to medical technologists to meet their precise educational requirements. 

The possible reasons for the poor pre-test performance can be lack of preparation for the virtual assessment, lack of hands-on activity earlier, especially for the junior cadre staff, and lack of periodic assessments on the usage of URT to keep them updated. The intervention based on synchronous and asynchronous activities proved fruitful, as depicted by the post-test scores using the same questionnaire. This highlights the utility of virtual platforms to supplement total quality management in the clinical laboratory.

Though COVID-19 affected all occupations with financial and emotional difficulties, medical technologists suffered from unprecedented challenges and fear due to the pandemic, including dissatisfaction about the measures taken by the management during the outbreak [10].

Hence, it is imperative to engage the historically neglected group with such interventions. This study provided an opportunity to engage medical technologists in pedagogy during challenging times while also improving the overall educational status as well as the laboratory's QC. In this regard, the administration of this education and evaluation intervention served a twin purpose during extraordinary times for healthcare professionals.

There were certain limitations to this study. The sample size was small and was also carried out at a single institute. However, this study was a first-of-itskind attempt to develop a refresher course for laboratory technologists in the region. The web-based assessment offered an inexpensive, self-administered tool with a rapid turnaround time and a high level of participation. Periodic conduction of such interventions will result in engagement, academic improvement, and better QC by the technologists hospital-wide.

The transition to synchronized online teaching and learning during the COVID-19 pandemic paved the path with a promising potential for the future of medical education for the overly occupied clinical laboratory scientists. This learning method is user-friendly, time-saving, and effective, as demonstrated by the results of this study.

## Dodatak

### Authors' Contribution

Sibtain Ahmed conceived the idea, designed the project, methodology, and research plan, searched the literature and drafted the manuscript. Muhammad Abbas Abid performed the data analysis and write-up of the work. Farhat Jahan developed the questionnaire, Google Forms, data collection and contributed to the intervention phase by conducting educational activities. Tomris Ozben provided supervision to the project and contributed to the discussion of the results and review and amelioration of the draft. All authors participated in the data discussion and revised the manuscript. All the authors have accepted responsibility for the entire content of this submitted manuscript and approved submission.

### Conflict of interest statement

All the authors declare that they have no conflict of interest in this work.

### List of Abbreviations

QC, quality control;<br>CAP, College of American Pathologists;<br>URT, Unity Real Time;<br>ILMS, Integrated Laboratory Management System;<br>COVID-19, Coronavirus disease 2019.
